# Improvement of straw decomposition and rice growth through co-application of straw-decomposing inoculants and ammonium nitrogen fertilizer

**DOI:** 10.1186/s12870-023-04254-3

**Published:** 2023-05-09

**Authors:** Wei Liu, Jichao Tang, Dahong Zhang, Xun Jiang, Bilin Lu, Wenjia Yang

**Affiliations:** 1 Hubei Collaborative Innovation Center for Grain Industry, Agricultural College, Yangtze University, Jingmi Road No. 88, Jingzhou, Hubei 434025 China; 2 Engineering Research Center of Ecology and Agricultural Use of Wetland, Ministry of Education, Jingzhou, Hubei 434025 China; 3 Hubei Provincial Key Laboratory of Waterlogged Disasters and Agricultural, Use of Wetland Yangtze University, Jingzhou, Hubei 434025 China

**Keywords:** Straw decomposition, Straw-decomposing inoculants, Ammonium bicarbonate, Rice yield

## Abstract

**Background:**

The growth of rice is reduced by the slow decomposition of accumulated straw, which competes with rice for soil nitrogen nutrient. In recent year, straw-decomposing inoculants (SDIs) that can accelerate straw decomposition and ammonium nitrogen (N) fertilizer that can quickly generate available N is increasingly adopted in China. However, it is still unknown whether the N demand of straw decomposition and crop growth can be simultaneously met through the co-application of SDIs and ammonium N fertilizer.

**Results:**

In this study, we investigated the effect of the co-application of SDIs and ammonium bicarbonate on decomposition rate of wheat straw, rice growth and rice yield over two consecutive years in rice-wheat rotation system. Compound fertilizer (A0) was used as control. The ratios of ammonium bicarbonate addition were 20% (A2), 30% (A3) and 40% (A4), respectively, without SDIs or with SDIs (IA2, IA3, IA4). Our results revealed that without SDIs, compared with A0, straw decomposition rate, rice growth and yield were improved under A2; However, under A3, rice yield was decreased due to the slow decomposition rate of straw and limited growth of rice during late growth stage. Combining SDIs and N fertilizer increased straw decomposition rate, rice growth rate and yield more than that of N fertilizer alone, especially under IA3. Compared with A0, straw decomposition rate, tiller number, aboveground biomass, leaf area index, root length, and nitrogen use efficiency were significantly increased by 16%, 8%, 27%, 12%, 17%, and 15% under IA3. Consequently, the average rice yield of IA3 was increased to 10,856 kg/ha, which was 13% and 9% higher, respectively, than of A0 and A2.

**Conclusion:**

Our results indicated that ammonium bicarbonate application alone carried a risk of nutrient deficiency during late growth stage and yield decline. Therefore, the co-application of SDIs and 30% ammonium N fertilizer substitution can be a favorable practice to simultaneously accelerate straw decomposition and increase rice crop growth.

## Background

Rice-wheat rotation is a major cropping system in the Yangtze River agricultural regions. These regions cover 13 million hectares and produce 72% of the total cereal yield in China [1, 2]. Recycling crop straw into the soil is common in rice-wheat rotation systems. Crop straw itself is rich in nitrogen, phosphorus, potassium and other nutrients necessary for crop growth. During the process of straw decomposition, these nutrients will be gradually released into the soil. Numerous studies have indicated that soil organic carbon content, the total and available contents of nitrogen, phosphorus and potassium can be significantly increased with straw decomposition; consequently, the fertility and physical properties of the soil can be greatly improved [3–6]. However, the accumulation of straw due to slow decomposition has adverse impacts on crop growth [7]. Accumulated straw makes tillage difficult and limits seedling and root growth. Pathogens, insect eggs, and weed seeds in accumulated straw also reduce crop growth [8, 9]. It is therefore desirable to accelerate the rate of straw decomposition in the soil to improve crop growth.

In recent year, incorporation of crop straw together with straw-decomposing inoculants (SDIs), a microbial preparation that accelerate straw decomposition, is being increasingly adopted in China [10]. SDIs are composed of enzymes and various microbial species, including fungi and bacteria, which quickly break down cellulose, hemicellulose, lignin, and other straw components into simple compounds that are rich in nutrients [11, 12]. Thus, SDIs addition is an effective measure to hasten the process of straw decomposition; however, the accelerated process will also bring other problems. For instance, the high C/N (carbon/nitrogen) ratio in crop straw drives microorganisms to absorb more N for decomposition process, and accelerating straw decomposition will increase the demand for more N to continue straw decomposition by SDIs. Therefore, larger amounts of N are required to support not only the higher rate of straw decomposition but also subsequent crop growth [13, 14]. If this N demand is not met, microorganisms will compete with crop for soil N nutrient, resulting in soil N deficiency [15, 16]. Nitrogen, as a critical macronutrient and a critical component of all proteins and nucleic acids, is essential to allow development of new plant cells and crop growth [17]. The N requirements of crop plants extend from seed development to seed harvested for yield. Therefore, crop growth is slowed, sometimes severely, when a N deficiency lowers protein levels and depresses cell function. It has been confirmed that the positive effect of SDIs on straw decomposition would be greatly weaken if the SDIs was applied alone without N fertilizer [18]. Therefore, a sufficient N supply is the premise of exerting the effect of SDIs to promote straw decomposition and crop growth.

The use of nitrogen (N)-containing compounds in fertilizer (N fertilizer) is a common practice to balance the soil C/N ratio during straw decomposition [19]. Rapid decay begins early after the straw is tilled into the soil. Both the N supply velocity and the rate of N production in this early stage are important for continued straw decomposition and later crop growth [20]. In the use of fertilizers, ammonium bicarbonate is dissolved in water to produce NH_4_^+^, and amide N fertilizers need to be broken down by microbial urease to produce NH_4_^+^ before they can be directly absorbed by crops [21]. Thus, ammonium N fertilizer can supply available N nutrient faster than amide N fertilizers, which suggests that N demand for rapid straw decomposition and faster crop growth early in the growing season could be quickly met if ammonium N fertilizer is used as basal fertilizer. It was reported that the competition for N between straw-decomposing microbes and crop growth was alleviated in earlier plant growth by substituting compound fertilizer with ammonium N [22, 23]. However, due to rapid nutrient loss, the use of ammonium N fertilizer as basal fertilizer carries the risk of nutrient deficiency in the subsequent growth stage, which results in yield decline.

It is still unknown whether the N demand of straw decomposition and crop growth can be simultaneously met through the co-application of SDIs and ammonium N fertilizer. Furthermore, an appropriate application ratio of ammonium N fertilizer also need to be determined to avoid N deficiency during late growth stage. In this study, we investigated different proportions of basal-applied ammonium bicarbonate-substituted fertilizer with or without SDIs to identify the optimal fertilization methods to simultaneously increase the decomposition rate of wheat straw and improve rice growth. The parameters measured were the variation of tiller number, the amount of aboveground biomass, leaf area index, and the extent of root growth during the different growth stages.

## Results

### Straw decomposition rate

**Fig. 1:**
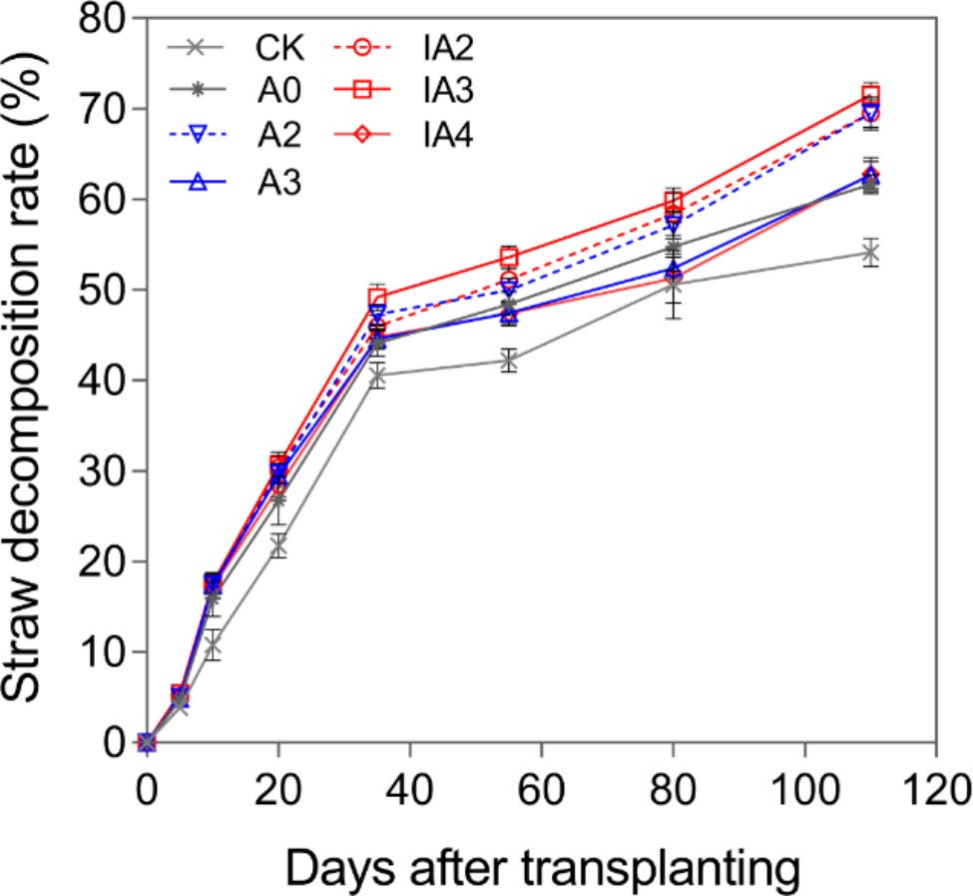
The variation of straw decomposition rate under different treatments in 2019

As shown in Fig. 1, the straw decomposition rate continually increased after the transplantation of the rice seedlings. From day 0 to day 35, the average decomposition rate of all treatments increased to 45.21%. However, from day 35 to day 110, the average decomposition rate of all treatments had only increased to 64.54%.

Straw decomposition rates differed among the treatments. The decomposition rates of A2, IA2, and IA3 were higher than those of the other treatments over the course of the experiment. IA3 produced the highest decomposition rate. Compared to A0, the decomposition rates across these treatments were significantly increased by 10–16% at day 35 and 110. There were small differences in the decomposition rates among A0, A3 and IA4.

### Tiller number and aboveground biomass

**Fig. 2:**
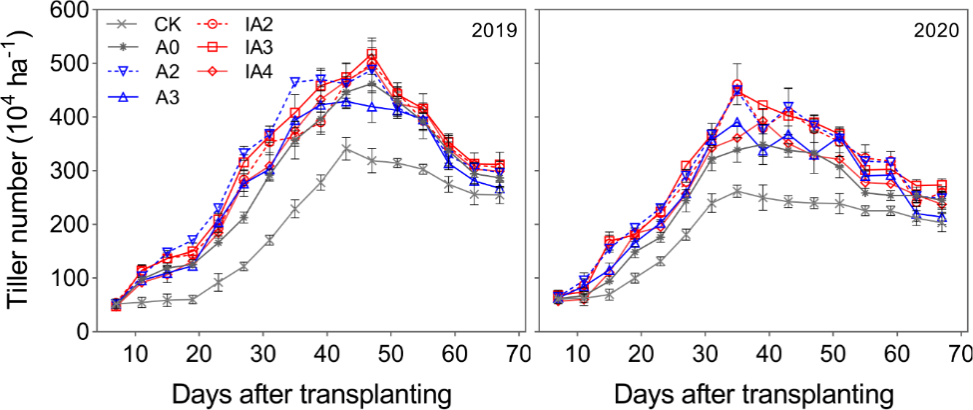
The variation of tiller number under different treatments in 2019 and 2020

The dynamic change in rice tiller number is shown in Fig. 2. The highest tiller number for each year was observed during days 35–47 post-transplantation. In 2019, the tiller numbers of A2, IA2, IA3, and IA4 were consistently higher than that of A0 after transplantation. Before day 39, IA2, IA3, and IA4 had significantly decreased tiller numbers compared to A2. However, after day 43, significantly increased tiller numbers were observed across these treatments. The tiller number of A3 significantly decreased after day 43 compared with A0. In 2020, A2, IA2, and IA3 had significantly increased tiller numbers compared to A0, and IA3 had the highest tiller number at day 77 post-transplantation. A3 and IA4 had significantly higher tiller numbers before day 59. However, after day 63, the tiller number of A3 significantly decreased and no further change in tiller number was observed for IA4 when compared with A0.

**Fig. 3:**
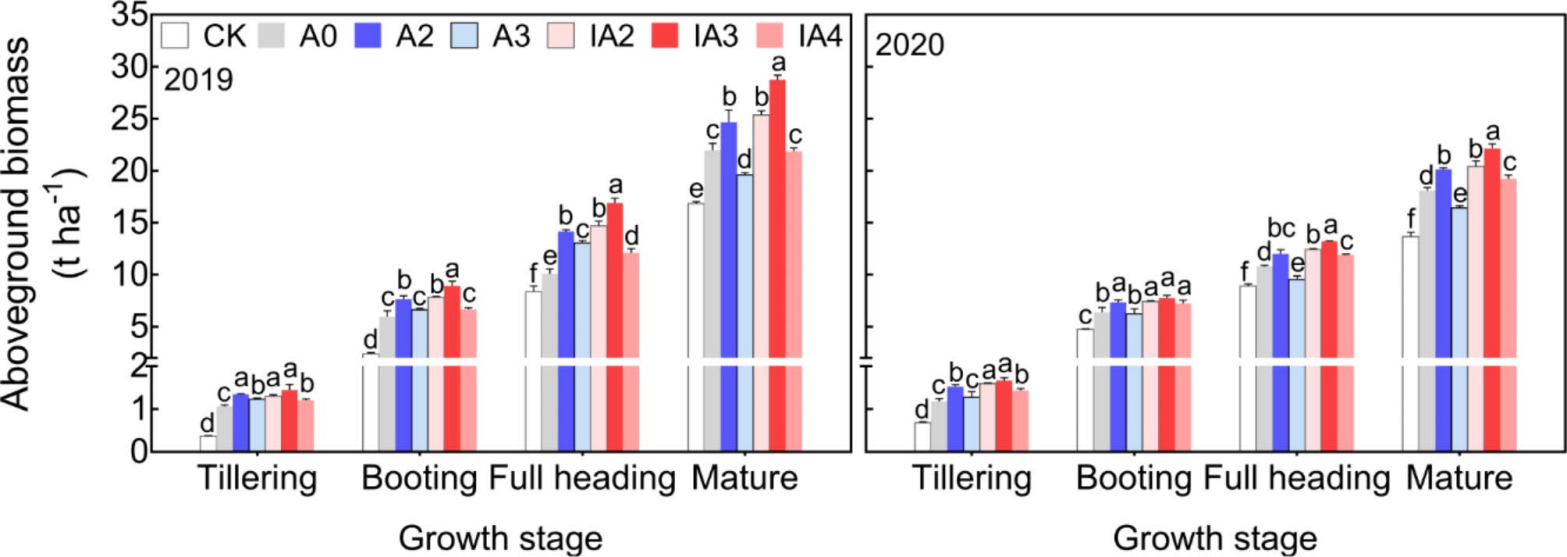
Aboveground biomass at different growth stages under different treatments in 2019 and 2020. Lowercase letters indicate significant differences among the seven treatments within the same growth stage *(p* < 0.05)

After the transplantation of rice seedlings, aboveground biomass increased and reached its highest values at the mature stage in each year (Fig. 3). In both years, from tillering to the mature stage, the aboveground biomass with IA3 was the highest among all treatments, followed by A2 and IA2. At the mature stage, the aboveground biomass of A2, IA2, and IA3 was significantly increased by 11–12%, 13–16%, and 22–31%, respectively, compared with A0. There was a small difference in the aboveground biomass between A3 and A0 before the booting stage. However, at the mature stage, the aboveground biomass of A3 was significantly lower than that of A0 by 9–10%. In 2020, treatment IA4 had significantly increased aboveground biomass compared to A0 at the mature stage.

### Leaf area index

**Fig. 4:**
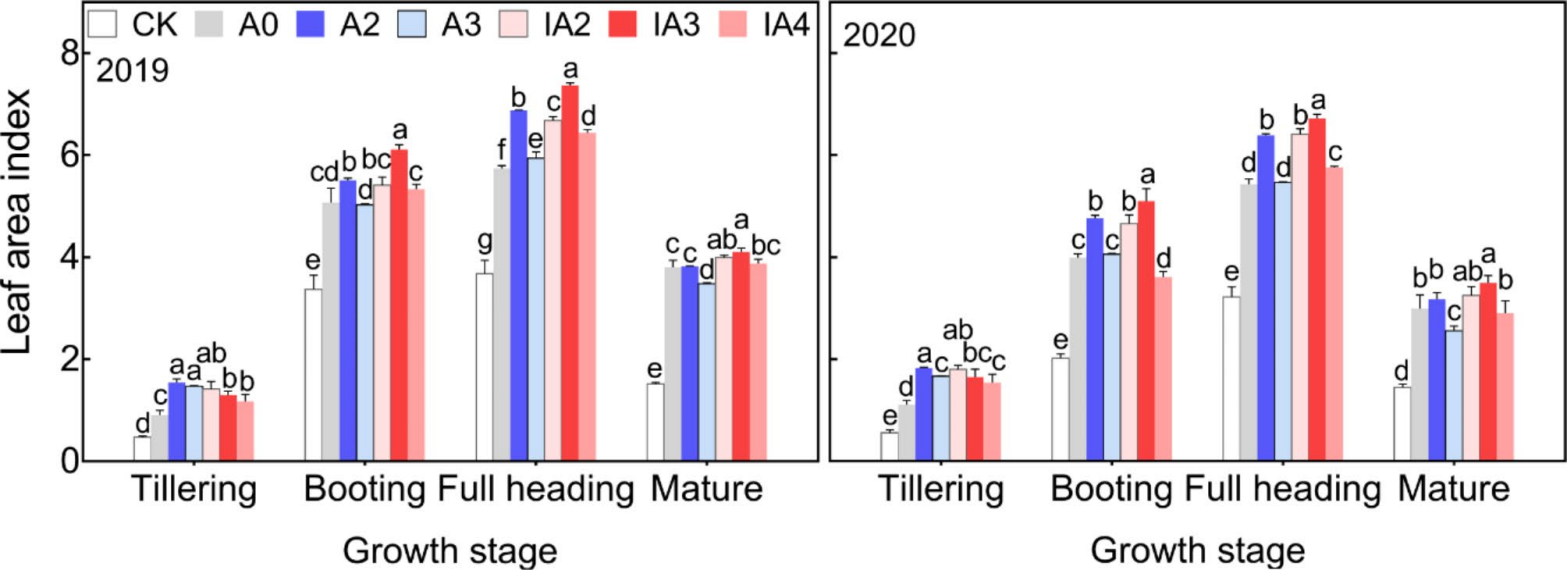
Leaf area index at different growth stages under different treatments in 2019 and 2020. Lowercase letters indicate significant differences among the seven treatments within the same growth stage *(p* < 0.05)

Leaf area index (LAI) was the highest at the full heading stage in each year (Fig. 4), and it differed among the different treatments throughout the growth stages. At the tillering stage, the LAIs of all treatments except for the CK were significantly higher than that of A0. At the booting stage, A2 and IA2 had significantly increased LAI in both years compared to A0. By contrast, the LAI of A3 showed no significant change compared with A0. At the full heading stage, the LAIs of A2, IA2, and IA4 in both years were significantly increased by 17–20%, 16–18%, and 6–12%, respectively, compared with A0. At the mature stage, we observed small differences in LAI among A0, A2, IA2, and IA4 in both years, and the LAI of A3 was significantly lower than that of A0. IA3 resulted in the highest LAI among the seven treatments after the booting stage. Compared with A0, the LAI of IA3 was significantly increased by 20–28%, 24–28%, and 8–17%, respectively, at the booting, full heading, and mature stages in 2019 and 2020.

### Root growth

**Fig. 5:**
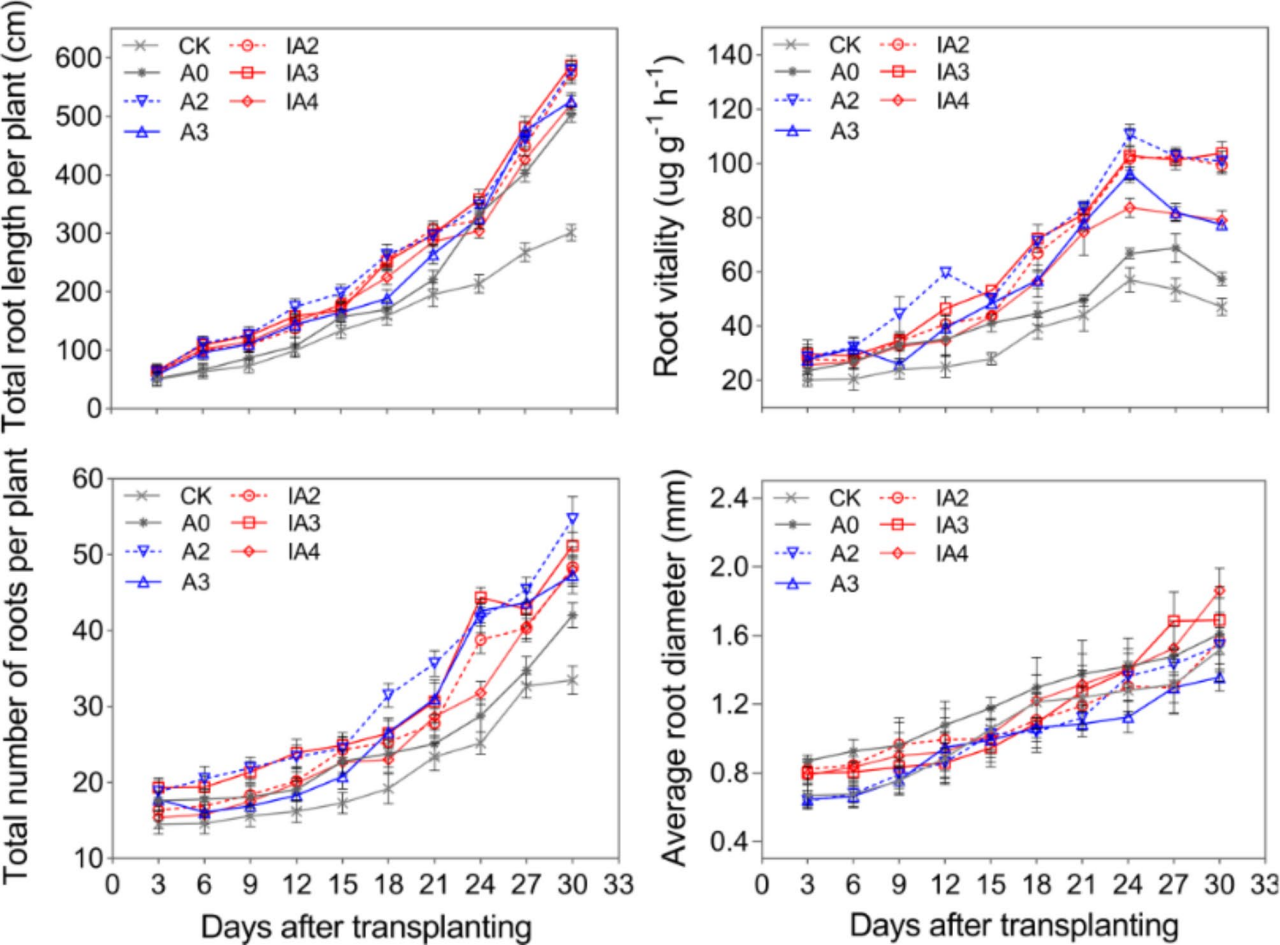
The variation of root growth under different treatments in 2019

The root length, root number per plant, and root diameter increased after transplanting. However, root vitality first increased and then decreased after day 24 post-transplantation (Fig. 5). Between 3 and 30 days after transplanting, the root length and root vitality of A2, IA2, and IA3 were consistently higher than those of other treatments. Compared with A0, the root length of A2, IA2, and IA3 was significantly increased by 4–69%, 11–51%, and 7–64%, respectively, and the root vitality was significantly increased by 20–76%, 2–73%, and 5–81%, respectively. Although the root length and root vitality of A3 and IA4 were lower than A2, IA2, and IA3, they were significantly higher than A0. All treatments (except the CK) had significantly increased root number compared to A0, with A2 showing the highest root number, followed by IA3; small differences in root number were detected among A3, IA2, and IA4. The root diameter of all other treatments was significantly lower than that of A0 before day 24, however, the root diameter of IA4 was significantly increased by 14% than A0 at day 30.

### N content in stem, leaf and grain of plants

**Fig. 6:**
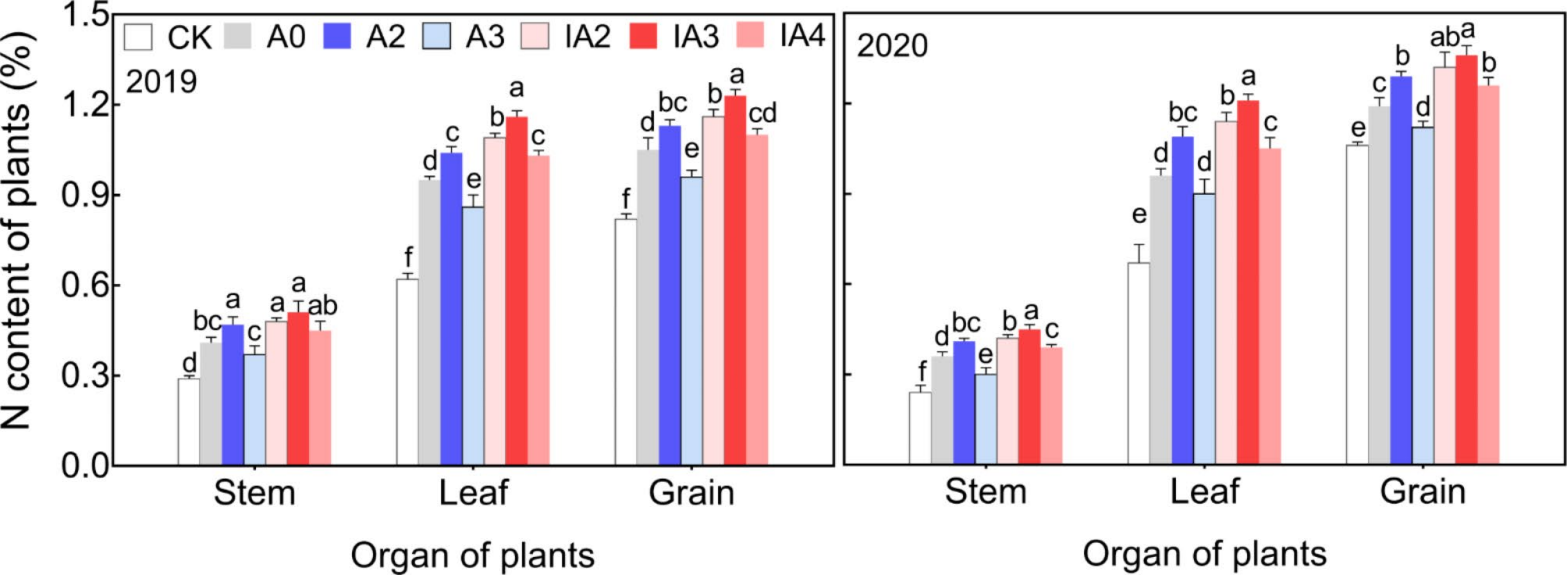
N content of plants at mature stage under different treatments in 2019 and 2020. Lowercase letters indicate significant differences among the seven treatments within the same organ *(p* < 0.05)

The change in N content in stem, leaf and grain at mature stage under different treatments is shown in Fig. 6. In stem, compared with A0, N content was significantly increased by 16–26% under A2, IA2 and IA3 in two years. N content in leaf was also significantly higher under A2, IA2 and IA3 than that under A0; in addition, IA4 had significantly increased N content in leaf compared to A0 in two years. In grain, N content was significantly increased by 8–17% under A2, IA2 and IA3 in 2019, and by 3–14% under A2, IA2, IA3 and IA4 in 2020 when compared with A0. Among the treatments (except CK), N contents in all organs was consistently lowest under A3.

### Nitrogen use efficiency

Nitrogen use efficiency was profoundly affected by the treatments in different years (Table 1). The total N content, ANUE, AE, and PFP_N_ of all treatments were significantly higher in 2019 than in 2020. In both years, the values of total N content, ANUE, AE, and PFP_N_ decreased with the following treatments: IA3 > IA2 > IA4 ≈ A2 > A0 > A3. Treatment IA3 had significantly increased total N content, ANUE, AE, and PFP_N_ by 8–9%, 19–20%, 44–51%, and 12–14%, respectively, compared to A0 and by 5–7%, 11–15%, 27–31%, and 8–9%, respectively, compared to A2. PNUE of the A3 and IA3 treatments was significantly higher than that of A0 in 2019, and in 2020, PNUE of all treatments (except CK) was significantly higher than A0.

**Table 1:** Total N content and nitrogen use efficiency under different treatments in 2019 and 2020

Year	Treatment	Total N (kg/ha)	ANUE (%)	AE (kg/kg)	PNUE (kg/kg)	PFP_N_(kg/kg)
2019	CK	113.20 ± 2.14f	/	/	/	/
	A0	200.25 ± 1.24d	44.64 ± 0.47d	14.45 ± 0.83c	32.37 ± 1.26c	52.54 ± 0.26d
	A2	204.23 ± 0.65c	46.68 ± 0.41c	15.86 ± 0.09c	33.98 ± 0.46bc	53.95 ± 0.13c
	A3	186.89 ± 0.85e	37.79 ± 0.40e	12.84 ± 0.16d	33.97 ± 0.28b	50.92 ± 0.20d
	IA2	210.58 ± 2.32b	49.93 ± 0.71b	17.02 ± 1.02b	34.09 ± 0.90c	55.11 ± 0.80b
	IA3	217.86 ± 0.59a	53.67 ± 0.80a	20.77 ± 0.41a	38.71 ± 0.42a	58.86 ± 1.30a
	IA4	203.50 ± 3.11 cd	46.31 ± 1.30 cd	15.48 ± 0.97bc	33.42 ± 1.38bc	53.56 ± 0.62c
2020	CK	104.79 ± 2.51f	/	/	/	/
	A0	180.41 ± 1.87d	38.78 ± 0.54d	12.94 ± 0.86c	33.36 ± 0.49c	45.90 ± 0.31d
	A2	186.51 ± 2.34bc	41.91 ± 1.15bc	15.40 ± 0.36b	36.75 ± 1.78b	48.36 ± 0.47b
	A3	169.39 ± 1.24e	33.13 ± 0.70e	12.27 ± 0.48c	37.04 ± 1.74b	45.23 ± 0.17e
	IA2	190.18 ± 2.04b	43.79 ± 0.78b	15.50 ± 1.28b	35.40 ± 0.31b	48.46 ± 0.57b
	IA3	197.64 ± 2.39a	47.61 ± 0.12a	19.53 ± 0.93a	41.01 ± 0.42a	52.49 ± 0.42a
	IA4	183.66 ± 2.06 cd	40.45 ± 0.32c	14.36 ± 1.35b	35.50 ± 0.58b	47.32 ± 0.23c
Year		**	**	*	ns	**
Treatment		**	**	**	**	**
Year * Treatment		**	**	**	*	*

*Note*: CF, compound fertilizer; AB, ammonium bicarbonate fertilizer; SDIs, straw-decomposing inoculants

### Yield and yield components

Rice yield significantly increased by 3–5% with A2 but decreased by 3% with A3 compared with A0. Rice yield of IA2 and IA3 significantly increased by 5–6% and 12–14%, respectively, compared with A0. The yield of IA4 was significantly lower than that of IA3 but significantly higher than that of A0 (Table 2). Both panicle number and filled-grain percentage were the highest with the IA3 treatment in both years, followed by A2 and IA2. The panicle number significantly increased by 14–21%, 5–15%, and 7–12%, respectively; the filled-grain percentage significantly increased by 8–11%, 4–9%, and 4–7%, respectively, under IA3, A2, and IA2 treatments compared with A0. In 2020, the number of spikelets per panicle and 1000-grain weight were significantly higher with the A3, IA2, IA3, and IA4 treatments compared with A0.

**Table 2:** Panicle number, spikelet per panicle, filled-grain percentage, 1000-grain weight and grain yield under different treatments in 2019 and 2020

Year	Treatment	Panicle (10^4^/ha)	Spikelet per panicle	Filled-grain percentage (%)	1000-Grain weight (g)	Grain yield (kg/ha)
2019	CK	229 ± 7f	181 ± 4b	76.89 ± 3.67d	26.78 ± 0.31c	7427 ± 142f
	A0	271 ± 10de	187 ± 2a	81.88 ± 0.89c	28.91 ± 0.16b	10,245 ± 50d
	A2	283 ± 2bc	172 ± 3c	85.00 ± 0.75b	29.53 ± 0.34a	10,520 ± 30c
	A3	266 ± 1e	186 ± 2ab	77.99 ± 2.88d	28.57 ± 0.24b	9930 ± 40e
	IA2	290 ± 5b	181 ± 3b	85.22 ± 1.28b	29.11 ± 0.40ab	10,746 ± 85b
	IA3	310 ± 2a	185 ± 3ab	88.17 ± 0.80a	29.19 ± 0.63ab	11,478 ± 158a
	IA4	278 ± 7 cd	190 ± 3a	78.63 ± 2.04d	28.89 ± 0.29b	10,445 ± 121c
2020	CK	180 ± 7d	143 ± 10d	71.08 ± 0.50d	28.07 ± 0.13e	6427 ± 143e
	A0	207 ± 10d	184 ± 2c	75.65 ± 0.96c	28.49 ± 0.15d	8950 ± 75d
	A2	238 ± 6b	183 ± 1c	80.37 ± 0.24a	29.63 ± 0.11ab	9430 ± 90b
	A3	213 ± 4c	206 ± 11a	75.11 ± 1.51c	28.65 ± 0.04d	8820 ± 150d
	IA2	231 ± 4b	192 ± 1b	78.56 ± 0.64b	29.89 ± 0.15a	9450 ± 22b
	IA3	252 ± 2a	200 ± 2a	81.39 ± 1.09a	29.30 ± 0.21c	10,235 ± 316a
	IA4	233 ± 6b	192 ± 1b	77.51 ± 1.22bc	29.47 ± 0.14bc	9227 ± 51c
Year		**	*	*	ns	**
Treatment		**	**	**	**	**
Year * Treatment		ns	ns	ns	ns	ns

*Note*: Different letters indicate significant differences among the different treatments in the same year (*p* < 0.05). * Significant at *p* < 0.05; ** Significant at *p* < 0.01; ns, not significant. ANUE, apparent nitrogen use efficiency; AE, agronomic NUE; PNUE, physiological NUE; PFP_N_, partial factor productivity of N

## Discussion

Our study showed that without SDIs, the straw decomposition rate and rice yield were higher with A2 but lower with A3 when compared with those with A0 (Fig. 1; Table 2). However, after adding SDIs, IA3 had significantly increased the straw decomposition rate and rice yield to 71.56% and 10,856 kg/ha, respectively, compared to A0 (61.64% and 9597 kg/ha, respectively) and A2 (69.52% and 9975 kg/ha, respectively). Furthermore, rice growth and NUE were also greatly improved with IA3 when compared with A2 and A0. The relatively poor performance of treatments lacking SDIs indicated that ammonium bicarbonate application without SDIs carried a risk of nutrient deficiency during the late growth stage and a decline in yield. However, the application of SDIs combined with ammonium bicarbonate-substituted compound fertilizer showed good performance in straw decomposition and crop growth. Therefore, it could be a desirable agronomic practice in rice fields when considering the dual goal of accelerating straw decomposition and offsetting straw decomposition-induced growth inhibition.

It was suggested that compared with the application of compound fertilizer alone, application of Ammonium N fertilizers can supply available N faster for the purpose of straw decomposition [21]. In this study, we showed that ammonium bicarbonate-substituted compound fertilizer (A2 treatment) significantly improved straw decomposition rate, consistent with previous observations [22, 23]. However, we also observed no increase in the straw decomposition rates of A3 and IA4 compared with A0, indicating that an excessive increase in the proportion of ammonium bicarbonate alone in basal fertilizer did not provide a long-term N source for straw decomposition. SDIs can degrade the cellulose, hemicellulose, and lignin in straw and improve quantity and activity of the soil microbial community, both of which contribute to straw decomposition [18, 24, 25]. Thus, the addition of SDIs to A2 should have increased straw decomposition rate. However, our results showed that the straw decomposition rate was not increased with IA2 (adding SDIs) compared to A2 (Fig. 1). We speculate that the N demand was not met under IA2 when 20% of the compound fertilizer was replaced by ammonium bicarbonate, because soil N will be consumed in large quantities to support straw decomposition and crop growth [13, 26]. However, treatment IA3 had significantly increased straw decomposition rate compared to A2 throughout the entire decay period, suggesting that SDIs combined with 30% ammonium bicarbonate-substituted compound fertilizer was more effective in promoting straw decomposition (Fig. 1). In general, it is crucial to select the suitable proportion of ammonium bicarbonate in basal fertilizer to accelerate straw decomposition after adding SDIs.

During the process of straw decomposition, microbial activity will compete with crop growth for soil N, resulting in soil N deficiency and crop growth inhibition, especially in the case of accelerated straw decomposition [15, 16]. In this study, however, treatments A2, IA2, and IA3 not only accelerated straw decomposition rate, but also improved tiller number, aboveground biomass, LAI, and root growth compared with A0 (Figs. 2, 3 and 4, and 5). The results indicated that ammonium bicarbonate substitution could alleviate growth inhibition induced by straw decomposition. Tiller number, LAI, and aboveground biomass with the A3 treatment increased at the early growth stage (tillering) compared with A0 but decreased in subsequent growth stages and became lower than A0 at maturity (Figs. 2 and 3, and 4). This may be due to the fast consumption of soil N by straw decomposition mediated by microbes and crop growth at early growth stages [21], resulting in insufficient nutrient supplies for subsequent growth.

Treatment IA3 had the highest tiller number, aboveground biomass, LAI, and root growth values among all treatments. Studies have proposed that including SDIs with fertilizer could accelerate straw decomposition, promote nutrient release from straw and improve soil fertility [3, 18, 25]. Furthermore, application of SDIs can also increase the quantity and activity of soil microbes [18, 24], improving the mineralization rate of soil organic matter, the structure of soil aggregates, and the physicochemical properties of soil [24, 27, 28]. The results of a meta-analysis of 1214 observations from 132 published studies showed that available N, P, K concentrations in the soil were significantly increased after the addition of SDIs [3]. In addition, soil-dissolved organic C also was increased through the application of SDIs [27, 29]. In this study, straw decomposition and crop growth performed best with IA3, indicating that the combined application of 30% ammonium bicarbonate-substituted fertilization and SDIs greatly improved the balance between the nutrient supply in the soil and the combined nutrient demand of straw decomposition and crop growth, which favored high crop productivity.

Root is the most important organ to absorb nutrients and water. Premature and quick leaf senescence was related with inadequate root number and root length [30]. Therefore, root development has a great influence on rice yield. It was reported that during the whole growing season, dry matter accumulation and translocation were profoundly affected by root activity, thus affecting grain yield [31]. Liu et al. [32] indicated that there was significant positive correlation between grain yield and both root number and root length. Nitika et al. [33] suggested that after transplanting, increasing root number is of great significance in providing yield stability and preventing yield reduction. In this study, both root vitality, root number, and root length were significantly higher under A2, IA2, and IA3 than those under A0; furthermore, rice yield was also significantly higher across these treatments. The results suggested that application of ammonium bicarbonate-substituted fertilization and SDIs was beneficial to root growth and development.

In this study, A2, IA2, IA3, and IA4 had significantly increased total N content, apparent NUE, agronomic NUE, physiological NUE, and PFP_N_ compared to A0 (Table 1). It was reported that NUE is greatly affected by the N source [34]. Thus, consistent with previous observation, NUE can be increased by combining different forms of N fertilizers when compared with the use of a single form of N fertilizer [35]. In addition, the total N content and NUE were the highest with the IA3 treatment, which may be the result of having the highest straw decomposition rate (Fig. 1). As we have noted, compared with A0, total N content and NUE were significantly decreased with A3 but were greatly increased with the co-application of SDIs with either 30% (IA3) or 40% ammonium bicarbonate (IA4) (Table 1). It has been proposed that *Bacillus*, especially *bacillus subtilis* and *bacillus licheniformis*, in straw-decomposing inoculants fix N [25], and that cellulolytic bacteria in straw-decomposing inoculants can inhibit ammonia volatilization and nitrate leaching by preventing the rapid transformation of chemical N fertilizer into NH_4_^+^ and NO_3_^−^ [36]. Upon the addition of SDIs, some N from ammonium bicarbonate might be fixed during the early growth stage and used by rice in subsequent periods, resulting in higher N content and NUE with IA3 and IA4.

## Conclusion

Our study demonstrated that both straw decomposition and crop growth can be significantly improved through the co-application of SDIs and ammonium N fertilizer. Without SDIs, increasing the proportion of ammonium bicarbonate in basal fertilizer could carry a risk of nutrient deficiency during the late growth stage, limiting crop growth and reducing yield, as with the A3 treatment. However, straw decomposition rate, crop growth (e.g., tiller number, aboveground biomass, leaf area index, and root growth), N use efficiency, and grain yield were all improved with IA3. Therefore, the co-application of SDIs and 30% ammonium bicarbonate-substituted fertilizer could prove to be a desirable agronomic method to accelerate the decomposition of straw and meet the N demand needed for maximal crop growth.

## Methods

### Experimental site

**Fig. 7:**
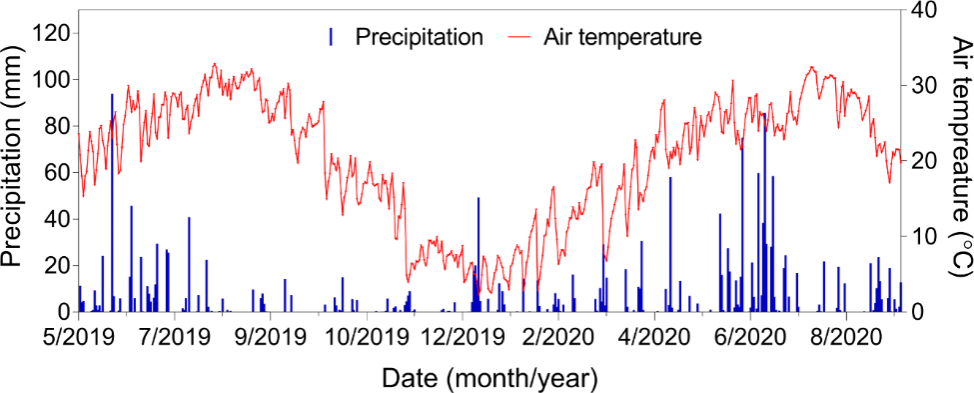
Daily variation of precipitation and air temperature in 2019 and 2020

A two-year field experiment was performed from May, 2019 to September, 2020 at the Yangtze University (30°22′N, 112°4′E), Jingzhou, Hubei province, China. The mean annual precipitation was 1100–1300 mm, the mean annual temperature was 15.9–16.6 °C, and the frost-free period was 242–263 days. In the experimental field, the soil texture was light loam (Kakingski soil texture classification system) with a pH of 7.89. At a 0–20 cm soil depth before the first sowing, the organic matter content was 15.83 g kg^− 1^, the alkaline N content was 44.3 mg kg^− 1^, the available phosphorus content was 19.7 mg kg^− 1^, and the available potassium content was 103.3 mg kg^− 1^. The daily variation of precipitation and air temperature are shown in Fig. 7.

### Experimental design and field management

**Fig. 8:**
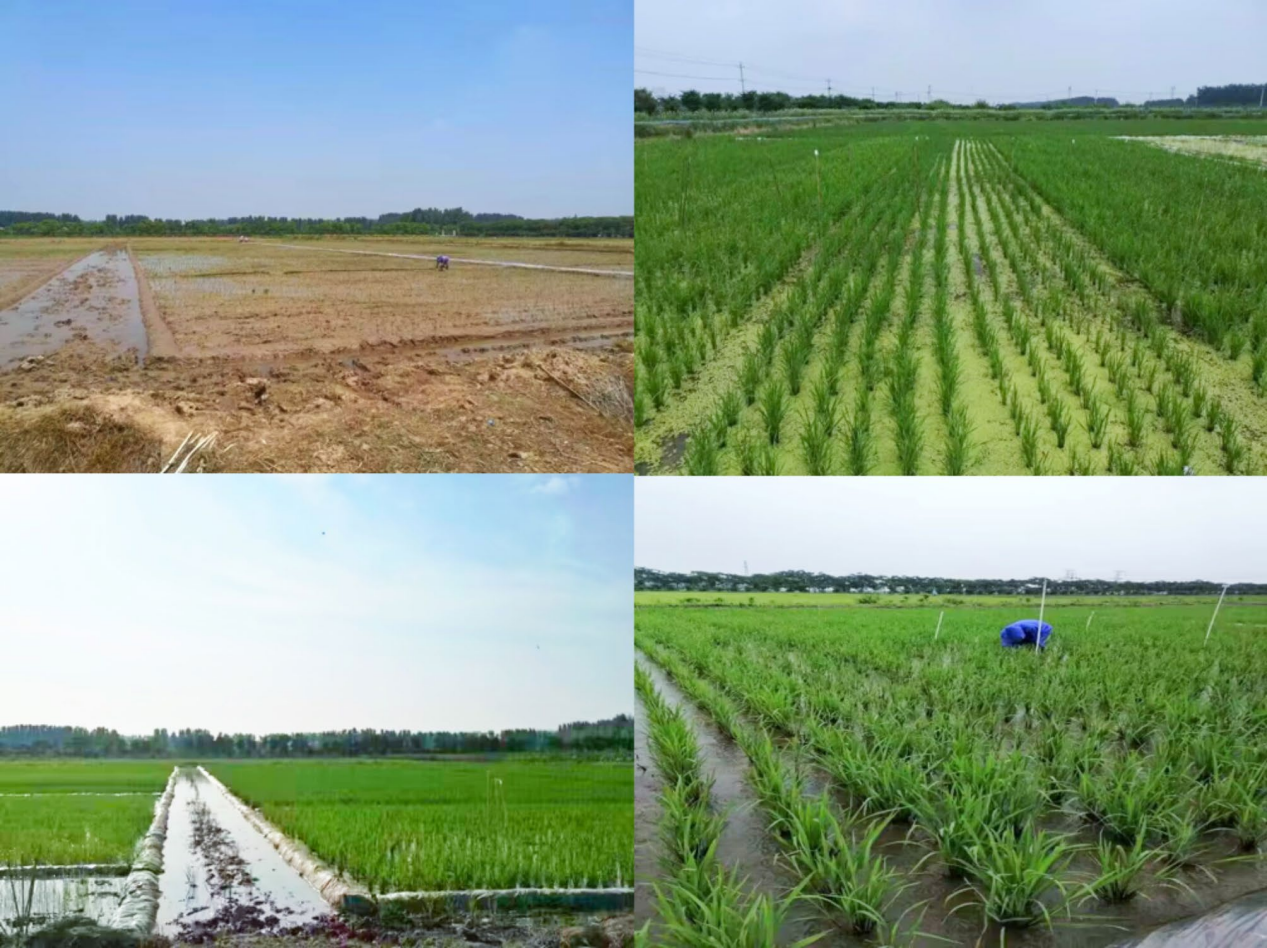
Photograph on this experiment in 2019

Seven treatments of different ratios of ammonium bicarbonate to compound fertilizer in the basal fertilizer with and without SDIs were applied to the crop fields, and the detailed treatments were listed in Table 3. The photograph on this experiment is shown in Fig. 8.

**Table 3:** The N application ratio/rate in different growth stages under each treatment

Treatment	Transplanting		Tillering (N ratio)	Panicle Initiation (N ratio)	N Rate (kg/ha)
	N ratio	SDIs (kg/ha)			
CK	0	0	0	0	0
A0	50%(CF)	0	30% (Urea)	20% (Urea)	195
A2	20% (AB) and 30% (CF)	0	30% (Urea)	20% (Urea)	195
A3	30% (AB) and 20% (CF)	0	30% (Urea)	20% (Urea)	195
IA2	20% (AB) and 30% (CF)	30	30% (Urea)	20% (Urea)	195
IA3	30% (AB) and 20% (CF)	30	30% (Urea)	20% (Urea)	195
IA4	40% (AB) and 10% (CF)	30	30% (Urea)	20% (Urea)	195

*Note*: Different letters indicate significant differences among the different treatments in the same year (*p* < 0.05). * Significant at *p* < 0.05; ** Significant at *p* < 0.01; ns, not significant

Each treatment was performed in triplicate, in a randomized complete block pattern. The area of each plot was 90 m^2^. The ridges (30 cm high and 30 cm wide) of each plot were covered by plastic film to prevent the movement of nutrients and water between adjacent plots. In both 2019 and 2020, rice (Quanliangyou 681) was cultivated in seedling trays from May 10 to May 13 and transplanted by a rice transplanter (Kubota Agricultural Machinery Co., Ltd., SPW-28 C) from June 1 to June 3. The rice was harvested from September 20 to September 23 in both years. The planting density in the field was 30 cm × 16 cm. The total N fertilizer application rate was 195 kg/ha in all treatments (except the CK treatment). Ammonium bicarbonate (17% N), compound fertilizer (N:P_2_O_5_:K_2_O = 15:15:15), and urea (46% N) were used as N fertilizer, which was split-applied at the ratio of 5:3:2 at the basal, tillering (7 days after transplantation), and panicle initiation stage (Table 3). The SDIs were produced by Shanghai Lianye Agricultural Science and Technology Co., Ltd., containing different fungi, bacteria and enzymes with ≥ 5 × 10^8^ CFU/g viable bacteria, ≥ 30 U/g cellulase activity and ≥ 30 U/g protease activity. The amount of wheat straw tilled into the soil was 4500 kg/ha each year. In addition, 97.5 kg/ha P_2_O_5_ and K_2_O were applied using calcium superphosphate (16% P_2_O_5_) and potassium chloride (60% K_2_O) in each treatment during transplanting.

### Measurement of the pH, the concentration of organic matter, alkaline N, alkaline phosphorus and the fast-acting potassium

Before the first sowing, we collected soil samples at a 0–20 cm soil depth from six spots randomly in our experimental field for determining the basic soil chemical properties. Soil pH was measured at a soil:water ratio of 1:2.5 (weight/weight) using a pH electrode [37]. Samples of the soil were ground to < 0.1 mm to allow for measurements of organic C using potassium dichromate volumetric method in conjunction with external heating method [38]. The soil alkaline N content was determined using the alkaline hydrolysis diffusion method [38]). Soil available phosphorus content was measured by treatment with 0.5 mol L^− 1^ NaHCO_3_ followed by molybdenum blue colorimetry [38]. Soil available potassium was extracted with 1 mol L^− 1^ NH_4_OAc (soil: solution 1:10) and determined using a flame photometer [38].

### Determination of the straw decomposition rate

The nylon mesh bag method was used to measure the straw decomposition rate [39]. Before transplantation, 5 cm segments of dried wheat straw (40 g) were placed in each of 120 mesh nylon bags (35 cm × 25 cm). Then, mesh bags were buried in each plot at a soil depth of 5–10 cm. At 5, 10, 20, 35, 55, and 80 days, a mesh bag was removed from plots, and the wheat straw was washed, dried, and weighed to determine the straw decomposition rate. The straw decomposition rate (%) was calculated as follows:

1 $${\rm{Straw}}\,{\rm{decomposition}}\,{\rm{rate}}\,\left( {\rm{\% }} \right)\,{\rm{ = }}\frac{{{{\rm{W}}_{\rm{0}}}{\rm{ - }}{{\rm{W}}_{\rm{n}}}}}{{{{\rm{W}}_{\rm{0}}}}}{\rm{ \times 100}}$$

where W_0_ is the initial dry weight of wheat straw and W_n_ is the dry weight of the remaining wheat straw.

### Measurement of the tiller number and aboveground biomass

After transplantation of rice seedlings, 10 representative plants (the plants and its surrounding plants grow normally) were randomly chosen in each plot for counting the variation of tiller number every four days. At the tillering (35 DAT), booting (60 DAT), full heading (80 DAT), and mature (110 DAT) stages, aboveground biomass was randomly sampled from the representative plants in each plot and weighed after oven-drying at 80 °C (until a constant weight was achieved).

### Measurement of the Leaf area index

Leaves from the representative plants were randomly collected in each plot at tillering, booting, full heading, and maturity stages. Leaf area was determined using ImageJ 1.51j8 (Wayne Rasband, NIH, USA). Leaf area index was calculated as the leaf area per plot divided by the land area per plot.

### Measurement of the Root length, number, diameter and vitality

After transplantation of rice seedlings, 10 representative plants were randomly selected every three days in each plot to determine total root length per plant, total number of roots per plant, average root diameter, and root vitality. A Vernier Caliper was used to measure root length and root diameter. The TTC (2,3,5-triphenyltetrazolium chloride) method was used to measure root vitality [40, 41].

### Determination of the nitrogen use efficiency

At maturity, the representative plants were randomly collected to determine the N content of the rice using the ECS 4024 CHNS-O Classic Analyzer (Costech, Italy). Apparent nitrogen use efficiency (ANUE, %), agronomic nitrogen use efficiency (AE, kg/kg), physiological nitrogen use efficiency (PNUE, kg/kg), and partial factor productivity of nitrogen (PFP_N_, kg/kg) were calculated according to the following equations [23]:

2 $${\rm{ANUE}}\,{\rm{ = }}\frac{{{{\rm{N}}_{\rm{1}}}{\rm{ - }}{{\rm{N}}_{\rm{0}}}}}{{\rm{N}}}{\rm{ \times 100}}$$

3 $${\rm{AE}}\,{\rm{ = }}\frac{{{{\rm{G}}_{\rm{1}}}{\rm{ - }}{{\rm{G}}_{\rm{0}}}}}{{\rm{N}}}$$

4 $${\rm{PNUE}}\,{\rm{ = }}\frac{{{{\rm{G}}_{\rm{1}}}{\rm{ - }}{{\rm{G}}_{\rm{0}}}}}{{{{\rm{N}}_{\rm{1}}}{\rm{ - }}{{\rm{N}}_{\rm{0}}}}}$$

5 $${\rm{PF}}{{\rm{P}}_{\rm{N}}}\,{\rm{ = }}\frac{{\rm{G}}}{{\rm{N}}}$$

where N is the total N fertilizer application rate (195 kg/ha); N_0_ and N_1_ are the total N content of plants in plots without and with N fertilizer application (kg/ha), respectively; and G_0_ and G_1_ are grain yield in plots without and with N fertilizer application (kg/ha), respectively.

### Measurement of the yield and yield components

At harvest time in each year, plants from the central 5 m^2^ in each plot were collected to determine the number of panicles, the number of spikelet per panicle, filled-grain percentage, 1000-grain weight, and grain yield.

### Statistical analysis

All data were analyzed using SPSS 20.0 Software (IBM, Chicago, IL, USA). To determine the significant differences among the different treatments, Duncan’s test (*p* < 0.05) was used to compare the means of the seven treatments. A mixed ANOVA model was used to test the significance of treatments, years, and the interaction of treatment × year.

## Data Availability

The datasets used and/or analysed during the current study available from the corresponding author on reasonable request.
